# Idiopathic Intracranial Hypertension: Prognostic Factors and Multidisciplinary Management

**DOI:** 10.1155/2017/5348928

**Published:** 2017-08-13

**Authors:** Claire Chagot, Marie Blonski, Jean-Loup Machu, Serge Bracard, Jean-Christophe Lacour, Sébastien Richard

**Affiliations:** ^1^Department of Neurology, Stroke Unit, University Hospital of Nancy, 54035 Nancy, France; ^2^Centre d'Investigation Clinique Plurithématique (CIC-P 1433), INSERM U1116, University Hospital of Nancy, 54500 Vandoeuvre-lès-Nancy, France; ^3^Department of Neuroradiology, University Hospital of Nancy, 54035 Nancy, France

## Abstract

**Background:**

Idiopathic intracranial hypertension (IIH) mostly affects young obese women and can lead to permanent visual impairment. However, prognostic factors and therapeutic strategy remain unclear.

**Methods:**

We retrospectively collected data from all patients diagnosed and managed for IIH in our university center from January 2001 to December 2016.

**Results:**

Seventy-nine patients were diagnosed with IIH. Bilateral transverse sinus stenosis (TSS) was found in 74% of the population. Visual outcome at 6 months was poor for 46% of patients, including all patients presenting weight gain of at least 5% since diagnosis (*p* < 0.001), whereas mean body mass index at diagnosis was not different between patients with poor versus good outcome (32.9 ± 7.7 versus 34.6 ± 9.4 kg·m^−2^). Other significant factors of poor prognosis were bilateral TSS (OR = 5.2; 95 CI: 1.24–24.9; *p* = 0.024). Thirteen patients with poor outcome after 6-month assessment underwent unilateral TSS stenting leading to visual improvement in 11 cases.

**Conclusion:**

Weight gain, rather than initial weight, emerged as the leading factor of poor visual outcome in patients with IIH, followed by presence of bilateral TSS. Consequently, first-line treatment must include dietary measures to control weight. Unilateral stenting appears to be a safe second-line treatment option for patients with bilateral TSS.

## 1. Introduction

Idiopathic intracranial hypertension (IIH) was first described by Dandy as “pseudo tumor cerebri” because of common clinical signs of intracranial hypertension without tumoral causes [1]. It predominantly affects young obese women and has an incidence of between 12 and 28 per 100,000 persons and per year [2, 3]. Although the population at risk and the clinical presentation seem to be homogeneous, the prognosis for each patient remains difficult to ascertain: some patients with IIH can suffer permanent visual impairment due to associated papilledema [4, 5]. However, the pathogenesis is poorly understood especially concerning transverse sinus stenosis (TSS), which could either be a cause of IIH or a consequence [6, 7]. Current patient management ranges from simple dietary measures to neurosurgical and endovascular procedures. Given this range of options, and in view of the risk of permanent visual impairment, there is a growing interest among clinicians to better understand IIH pathogenesis and identify prognostic factors so as to be able to propose an adapted management strategy.

We describe a population of IIH patients consecutively diagnosed and managed over a period of 15 years in our university center including the Departments of Neurology, Ophthalmology, Neurosurgery, and Interventional Neuroradiology to identify factors associated with poor visual outcome. From our experience and a literature review, we discuss the pathogenesis of IIH and propose a decision algorithm for therapy.

## 2. Materials and Methods

### 2.1. Patient Inclusion

We conducted an observational, retrospective study of patients diagnosed with IIH in the University Hospital of Nancy from January 1, 2001, to December 31, 2016. The patients were identified from our database through the terms: “idiopathic intracranial hypertension”, “benign intracranial hypertension”, “empty sella”, and “distension of the perioptic subarachnoid space”. All patients over 16 years with a diagnosis of IIH according to the modified Dandy criteria were included (Table 1) [8]. In accordance with data from literature, we also retained patients with probable diagnosis presenting criteria A to D [9, 10]. Patients with secondary intracranial hypertension (due to hormonal disorders or medical treatment as steroids and cyclins) were excluded.

### 2.2. Data Collection

Demographic data (sex, age), clinical signs (ophthalmic and neurological symptoms, time to diagnosis, body mass index (BMI), and CSF opening pressure), radiological signs on cerebral MRI (empty sella, distension of the perioptic subarachnoid space, and presence of TSS on time-resolved imaging of contrast kinetics (TRICKS)), and therapeutic strategies used (medical, neurosurgical, and endovascular) were collected.

Outcome at 6 months was assessed from ophthalmic examination with visual acuity, visual field, and fundus exams. Good outcome was defined as visual improvement and papilledema resorption and poor outcome as persistence or worsening of papilledema and/or visual field. Changes in weight from diagnosis to 6-month assessment was also taken into account and classified as weight loss (>5% of initial bodyweight), weight gain (>5%), or steady weight (<5%).

Long-term outcome (after 6 months) for all patients was also collected and assessed from fundus exams (papilledema or not) at the last follow-up visit.

### 2.3. Statistical Analysis

Continuous variables were reported as median (range) or mean ± standard deviation. Categorical variables were reported as frequency and percentage. A first statistical comparison was performed for all collected criteria between patients with good versus poor visual outcome, using Student's *t*-test and Fisher's exact test when appropriate. We retained criteria found significant at the *p* < 0.05 level to perform logistic regression in order to compute odds ratio (OR), OR 95% confidence interval (95 CI), and *p* value. Statistical analyses were performed using SAS version 9.4 (SAS Institute Inc., Cary, NC, USA).

### 2.4. Ethics

The study received the required legal approval from the appropriate French Data Protection Committee (Commission Nationale de l'Informatique et des Libertés) number 2017438v0.

All data were anonymized before the analyses were conducted.

## 3. Results

Seventy-nine patients (73 females and 6 male) were diagnosed with IIH in our center during the study period. Overall population characteristics are described in Table 2. Mean age at diagnosis was 33 years ranging from 16 to 63. Median time from first symptoms to diagnosis was 2 months (range: 1–48 months). Mean BMI at diagnosis was 35 ± 9.7 kg·m^−2^ (range: 17–68.7 kg·m^−2^) and 69% of patients had a BMI > 30 kg·m^−2^.

The most frequent clinical signs were headache found in 82% of patients, papilledema (bilateral in all cases) in 96%, and visual field loss in 87%.

Median CSF opening pressure was 285 mm CSF (range: 150–540 mm CSF) and 18 (33%) patients presented a pressure below 250 mm CSF.

**Figure 1:**
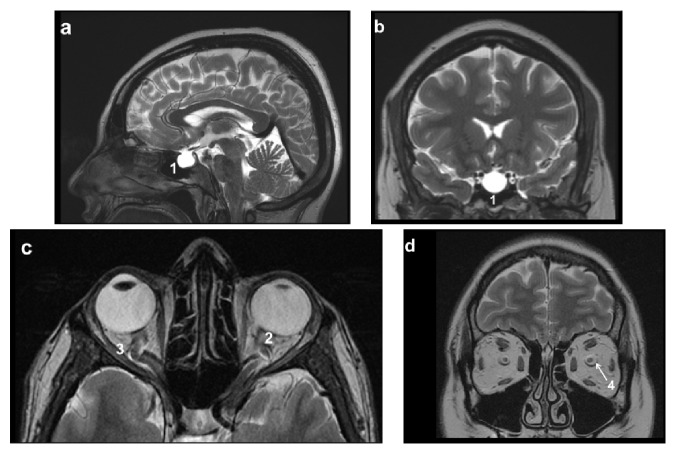
Signs of idiopathic intracranial hypertension on cerebral MRI, T2-weighted images. 1: empty sella, 2: flattening of the posterior aspect of the globe, 3: tortuous optic nerve, and 4: distention of the perioptic subarachnoid space.

**Figure 2:**
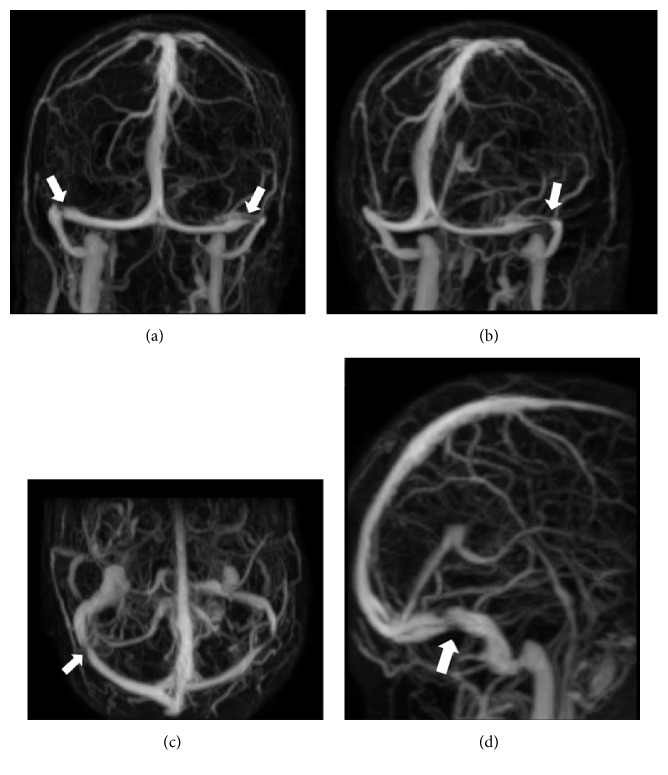
Bilateral transverse sinus stenosis on cerebral MR angiography; time-resolved imaging of contrast kinetic. Arrows: transverse sinus stenosis.

Cerebral MRI showed empty sella and distension of the perioptic subarachnoid space in 57% and 65% of cases, respectively (Figure 1). TRICKS were performed in 73% of the cases in which unilateral TSS or hypoplasia was found in 17% and bilateral TSS (bilateral stenosis or unilateral stenosis and hypoplasia) in 74% (Figure 2).

Before the 6-month assessment, 91% of the patients received a median daily dose of 623 mg of acetazolamide. Weight loss was achieved in 31% of the patients and steady weight in 48%, whereas 21% presented weight gain.

At the 6-month assessment, poor visual outcome was observed in 46% of documented cases (Table 2). Outcome was not assessable for 12 patients due to loss of follow-up or missing data. No statistical difference was found between the groups of patients presenting poor versus good visual outcome concerning gender, age, BMI, and clinical characteristics at the initial presentation. All the patients with a weight gain of at least 5% at 6 months presented poor outcome (*p* < 0.001) with a median BMI increase of 5.2 kg·m^−2^. The only other sign found significantly more frequently in the group with poor outcome was bilateral TSS (OR = 5.2; 95 CI: 1.24–24.9; *p* = 0.024).

In the subgroup of patients presenting bilateral TSS only, those with poor outcome were significantly older (37 ± 11 versus 30 ± 11 years; OR = 1.07; 95 CI: 1–1.15; *p* = 0.043). Overall, median CSF pressure at diagnosis tended to be higher in the group with poor outcome (305 versus 260 mm CSF) and for patients with bilateral TSS only (297 versus 262 mm CSF) but without reaching significance. There was no significant difference in patients who presented bilateral TSS versus those who did not, for any of the criteria including CSF opening pressure.

**Figure 3:**
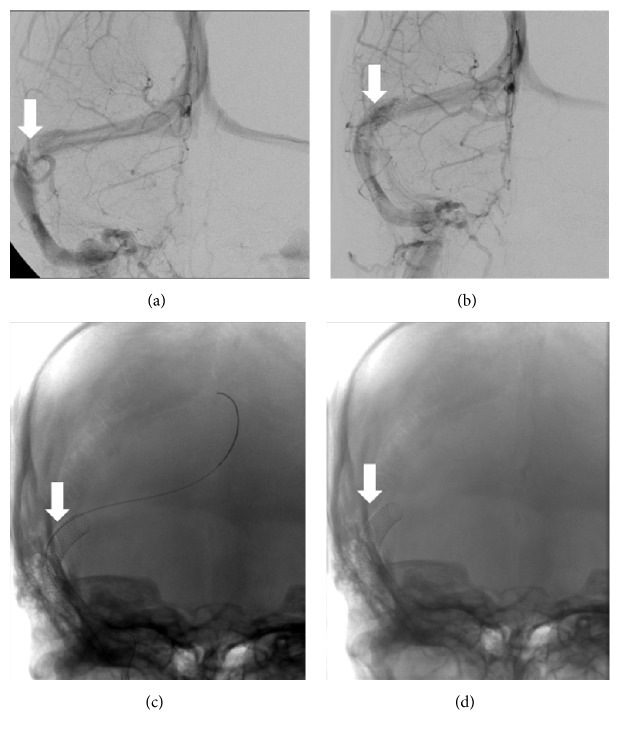
Stenting of right transverse sinus stenosis in a patient with bilateral stenosis, conventional angiography. (a) Right transverse sinus stenosis, (b) condition after treatment, and (c, d) stent placement.

Regarding long-term outcome, no papilledema recurrence was observed in the patients with good outcome at 6 months with a median time of follow-up of 8 months. Resorption of papilledema was obtained in 18 of the 31 patients with poor outcome at the 6-month assessment after a median follow-up time of 27 months. Among them, three patients underwent bariatric surgery resulting in weight loss. TSS stenting was performed in 13 patients and resulted in good outcome in all but two (Figure 3): one patient presented stent thrombosis requiring transient anticoagulation therapy and CSF diversion, and the other one required bariatric surgery because of continuous weight gain. Both of these patients finally achieved good outcome. One patient presented a right femoral artery pseudoaneurysm due to the TSS stenting procedure. Another underwent successive optic nerve sheath fenestration (ONSF) and CSF diversion without improvement requiring TSS stenting which resulted in good outcome. A third patient underwent CSF diversion after medical treatment but was lost to follow-up.

## 4. Discussion

Our results indicate that the first reason for poor IIH prognosis is weight gain followed by bilateral TSS. This suggests an interaction of these major factors in the pathogenesis of IIH. IIH is primarily diagnosed by Dandy's criteria revised by Friedman et al. [8], although some authors retain a diagnosis of IIH even if the CSF opening pressure does not reach 250 mm CSF [4, 10]. Furthermore, diagnosis is mainly based on clinical arguments whereas improvement in papilledema under treatment should also be taken into account [9, 11]. IIH affects almost exclusively young obese women. In our region (Lorrain, France), the prevalence of obesity has increased by 62% over the last 15 years and represented 17% of the general population (especially young women from 25 to 34 years) in 2012 [12]. A corresponding increase was observed in the incidence of IIH in our center which underlines the involvement of a high BMI in the development of the disease [13]. The hypothesis is that increased abdominal mass is responsible for elevated intrathoracic pressure resulting in compromised venous return from the head and neck [14]. This hypothesis is backed up by clinical improvement after weight loss [15, 16]. However, while morbid obesity (BMI > 40 kg·m^−2^) has already been demonstrated as a factor of poor visual outcome [17], initial BMI was not different between the patients with good and poor visual outcome in our study. We suggest therefore that the leading factor is weight gain rather than initial BMI, which could partially explain the low incidence of IIH in the obese population and the lack of correlation between CSF opening pressure and BMI [5, 18–20]. Cytokines, and in particular adipokines which are specifically produced by adipose tissue, have become a research focus. They are thought to increase CSF secretion via ions and water change across Na+/K+ ATPase in the choroid plexus cells [21]. Significantly high levels of leptin, a product of the obese gene involved in weight homoeostasis, have been found in the CSF of IIH patients [22]. Sinclair et al. also suggested a role of 11*β*-hydroxysteroid dehydrogenase type 1, an enzyme regulating CSF production through the glucocorticoid signaling pathway [23]. An increase of the water channel aquaporin 1 in choroid plexus is also suggested to promote raise of intracranial pressure [24]. IIH is clearly predominant in women but, to date, no sex-dependent hormonal profile has been identified as participating in its pathogenesis [25].

**Figure 4:**
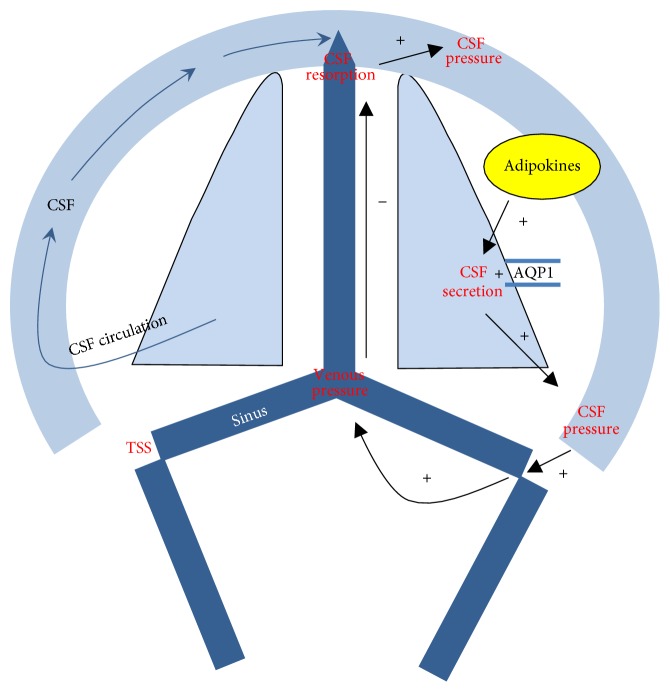
Proposed mechanisms for idiopathic intracranial hypertension pathogenesis. AQP1: aquaporin 1, CSF: cerebrospinal fluid, and TSS: transverse sinus stenosis.

TSS was frequent in our population, similarly to the series reported in literature (up to 90% of the cases), and is considered to be a marker of IIH [6, 26–28]. This anatomic condition raises two important issues: whether it is of constitutional or acquired nature and what role it plays in the pathogenesis of IIH. The foremost theory is that TSS results from collapse of the cerebral venous system under high pressure. Some studies describe TSS regression after CSF diversion or evacuation [7, 29, 30]. However, similarly to other studies, we did not find a significantly higher CSF opening pressure in patients with bilateral TSS [31]. TSS has also been suggested to be the one of the primary causes of IIH because of its persistence after normalization of CSF pressure [32] or a decrease in the venous pressure gradient [33–35]. Moreover, a significant decrease in CSF opening pressure has been observed after unilateral TSS stenting [36]. Finally, we can hypothesize that bilateral TSS and intracranial pressure are related [29]. Intracranial hypertension promotes the collapse of the transverse sinus resulting in increased venous pressure and impairment of passive CSF resorption (Figure 4).

Our series is characterized by a high rate of poor visual outcome at 6 months, possibly related to the high percentage of patients with observed weight gain at the 6-month assessment [5, 37, 38]. An increase in weight emerged as the leading criterion for outcome followed by presence of bilateral TSS. In the literature, other factors such as male gender, black race, younger age of onset, high CSF opening pressure, more severe obesity, and papilledema have been identified as affecting outcome to various degrees [39–45]. Some specific ophthalmologic factors (thinner retinal ganglion cell and inner plexiform layer complex and optic disc hemorrhage) have also been reported [44, 46]. We are the first to identify bilateral TSS in the whole population and an older age in patients with bilateral TSS, as a factor of poor outcome. The latter scenario could be due to persistence of stenosis in a patient with longstanding IIH.

**Figure 5:**
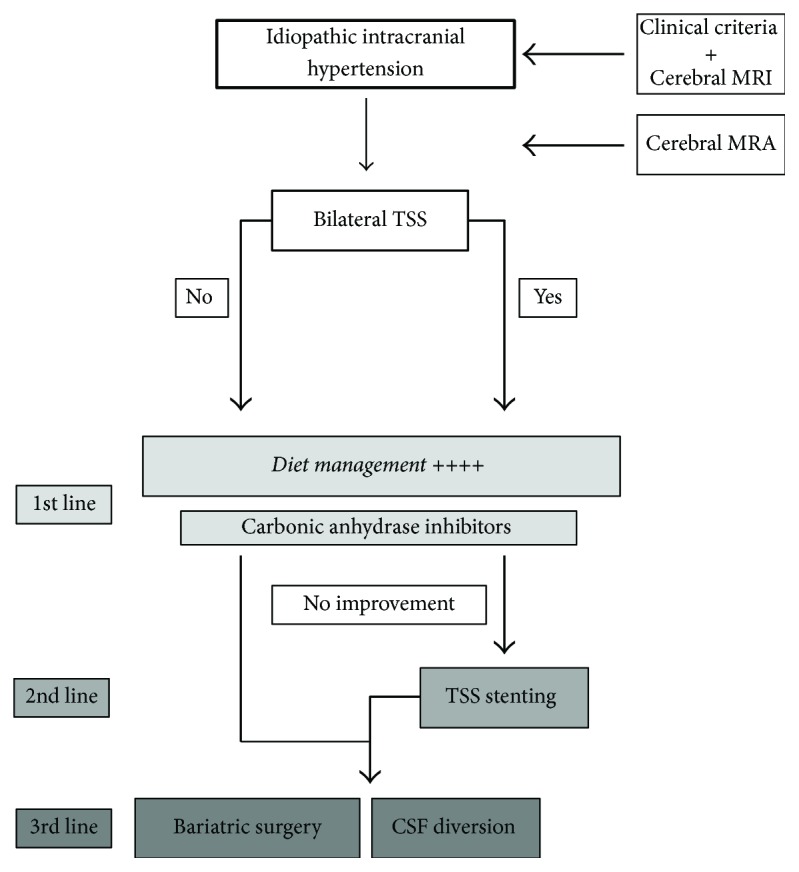
Algorithm for management of patients with idiopathic intracranial hypertension. CSF: cerebrospinal fluid, MRA: magnetic resonance angiography, and TSS: transverse sinus stenosis.

The main goal of therapeutic strategies is to decrease intracranial pressure in order to prevent visual impairment due to papilledema. Management strategies have mainly been based on clinical experience but prospective trials assessing medical care have recently been published [15, 36, 47, 48]. In view of our results, weight control is crucial when managing a patient with IIH. Significant IIH improvement and reduction in intracranial pressure have already been demonstrated to be correlated with weight loss in a prospective cohort [15]. This critical outcome of weight loss was only achieved in some of our patients despite dietary recommendations during neurological follow-up. Given the importance of this factor, all patients with IIH should be systematically managed by a nutritionist. Bariatric surgery with fast weight reduction resulted in good outcome in some of our patients [49]. However, this procedure should only be considered as a second-line therapy because of the high complication rate (incisional hernia, stenoses, ulcers, and nutrition deficiency) [50, 51] and long treatment time including physical and psychological monitoring [52]. Carbonic anhydrase inhibitors, which reduce CSF secretion via inhibition of ions and water movement across the plexus choroid, are also used to manage IIH. High doses of acetazolamide (4 g per day) have been shown to significantly improve visual impairment in a double-blind placebo-controlled trial [48]. However, once again, the study did not demonstrate a medication effect independent of weight loss. Reported adverse events were frequent including paresthesia, fatigue, and dysgeusia. Topiramate, a weaker carbonic anhydrase inhibitor than acetazolamide, also promotes weight loss and conveys a comparable effect on visual outcome as acetazolamide but with fewer adverse events [53]. If medical strategies are unsuccessful, then surgical and endovascular procedures can be an option (Figure 5). Nevertheless, none of these have been assessed through randomized clinical trials. A systematic review has reported that ONSF and CSF diversion improve papilledema in about 80% and 70% of cases, respectively, but with a high rate of complications (mainly local complications for ONSF and infections, subdural hematomas, and shunt revision for CSF diversion) [34]. In this context, TSS stenting would seem to be a safer option with papilledema resorption and visual improvement achieved for 80 to 97% of cases after unilateral stenting [33–36]. Fewer than 3% of patients experience complications but additional procedures are required in 10%, mainly for adjacent restenosis. Procedure failure is more frequently reported in patients with a high CSF opening pressure [54], and stent replacement is more often required in those with a high cerebral venous pressure gradient and bilateral TSS [35, 55]. One patient in our series presented a stent thrombosis without any identified thrombophilia, which represents an unusual complication [56].

A few limitations of our study deserve to be mentioned. The main one is due to its retrospective nature and the amount of missing data. Furthermore, bilateral TSS as a prognostic factor has not been demonstrated to date in literature so our findings will need to be confirmed in multicentric prospective studies [57]. These future works are likely to identify other interesting criteria such as CSF opening pressure, which did not reach significance in our study. Moreover, TSS stenting, which represents one of the most interesting second-line treatments, also deserves to be prospectively assessed.

## 5. Conclusions

In patients with IIH, weight gain, as opposed to initial BMI, is the leading factor of poor visual outcome. Other identified criteria, but to a lesser extent, are bilateral TSS in the overall population and older age in patients with bilateral TSS only. This emphasizes cross-links between weight gain and high cerebral venous pressure in the pathogenesis of IIH. Unilateral TSS stenting appears to be a safe and effective treatment to overcome this pathological circle in patients with bilateral TSS but should be reserved only after measures are undertaken to achieve weight loss.

## Figures and Tables

**Table 1:** Diagnosis of idiopathic intracranial hypotension from Dandy's criteria modified by Dandy et al. [1, 8].

*Required for diagnosis of IIH*	

*A*	Papilledema
*B*	Normal neurologic examination except for cranial nerve abnormalities
*C*	Neuroimaging: normal brain parenchyma without evidence of hydrocephalus, mass, or structural lesion and no abnormal meningeal enhancement on MRI
*D*	Normal CSF composition
*E*	Elevated lumbar puncture opening pressure (>250 mm CSF in adults) in a properly performed lumbar puncture

*Diagnosis of IIH without papilledema*	

In the absence of papilledema, a diagnosis of pseudotumor cerebri syndrome can be made if *B–E above are satisfied,* and *in addition the patient has a unilateral or bilateral abducens nerve palsy*	
In the absence of papilledema or sixth nerve palsy, a diagnosis of pseudotumor cerebri syndrome can *be suggested but not made if B–E above are satisfied,* and *in addition at least 3 of the following neuroimaging criteria are satisfied*	
*1*	Empty sella
*2*	Flattening of the posterior aspect of the globe
*3*	Distention of the perioptic subarachnoid space with or without a tortuous optic nerve
*4*	Transverse venous sinus stenoses

IIH: idiopathic intracranial hypotension.

**Table 2:** Population characteristics and comparison of good versus poor 6-month visual outcome.

Criteria	Total population *n* = 79	Good outcome *n* = 36	Bad outcome *n* = 31	*p*
*Sex*				1^a^
Female, *n* (%)	73/79 (92.4)	33/36 (91.7)	28/31 (90.3)	
Male, *n* (%)	6/79 (7.6)	3/36 (8.3)	3/31 (9.7)	

*Age* years, mean ± SD	33 ± 12	31.5 ± 12.7	35 ± 11.4	0.22^b^

*Time to diagnosis*, months, median (range)	2 (1–48)	4.7 (1–48)	2.8 (1–8)	0.30^b^

*BMI at diagnosis*, kg·m^−2^, mean ± SD	35 ± 9.7	34.6 ± 9.4	32.9 ± 7.7	0.43^b^
BMI < 30, *n* (%)	21/68 (30.9)	11/32 (34.4)	9/28 (32.1)	0.57^a^
BMI 30–35, *n* (%)	13/68 (19.1)	5/32 (15.6)	8/28 (28.6)	
BMI 35–40, *n* (%)	11/68 (16.2)	5/32 (15.6)	4/28 (14.3)	
BMI > 40, *n* (%)	23/68 (33.9)	11/32 (34.4)	7/28 (25)	

*CSF opening pressure* mmCSF, median (range)	285 (150–540)	260 (170–420)	305 (150–540)	0.06^b^

*Clinical signs*				
Headache, *n* (%)	65/79 (82.3)	27/36 (75)	27/31 (87)	0.23^a^
Papilledema, *n* (%)	75/78 (96)	35/36 (97)	31/31 (100)	1^a^
Visual acuity, median, /10	8.25	8.5	8.3	0.33^a^
Transient visual obscuration, *n* (%)	14/79 (17.7)	5/36 (13.8)	5/31 (16.1)	1^a^
Visual field defect, *n* (%)	62/71 (87.3)	28/32 (87.5)	26/28 (93)	0.67^a^
Eye-tracking impairment, *n* (%)	12/79 (15.2)	9/36 (25)	3/31 (9.7)	0.12^a^
Tinnitus, *n* (%)	10/79 (12.7)	4/36 (11)	6/31 (19.3)	0.49^a^
Dizziness, *n* (%)	9/79 (11.4)	3/36 (8.3)	6/31 (19.3)	0.28^a^

*Radiological signs*				
Empty sella, *n* (%)	45/79 (57)	22/36 (61)	19/31 (61.3)	1^a^
Optic nerve sheath enlargement, *n* (%)	51/79 (64.6)	21/36 (58.3)	22/31 (71)	0.32^a^

*Transverse sinus*				0.024^a^
Normal, *n* (%)	5/58 (8.6)	3/26 (11.5)	0/28	
Hypoplasia, *n* (%)	5/58 (8.6)	3/26 (11.5)	2/28 (7.1)	
Unilateral stenosis, *n* (%)	5/58 (8.6)	4/26 (15.5)	1/28 (3.6)	
Bilateral stenosis, *n* (%)	43/58 (74)	16/26 (61.5)	25/28 (89.3)	

*Treatments*				
Acetazolamide daily dose, mg, median	623	640	651	0.87^b^
Weight change				<**0.00**1^a^
Gain > 5%, *n* (%)	10/48 (20.8)	0/26	10/22 (45.5)	
Loss > 5%, *n* (%)	15/48 (31.2)	11/26 (42.3)	4/22 (18.2)	
No change, *n* (%)	23/48 (48)	15/26 (57.7)	8/22 (36.3)	

BMI: body mass index; CSF: cerebrospinal fluid; SD: standard deviation; a: Fisher's exact test, b: Student's *t*-test; *n*: number of patients, presented as number of events/number of documented cases (and percentage). 12 patients were lost to follow-up or missed data.
